# Seasonal temperature fluctuation and snail adaptive behaviors yield insights into the dynamics and distribution of schistosomiasis in Africa

**DOI:** 10.21203/rs.3.rs-6264426/v1

**Published:** 2025-03-26

**Authors:** Ibrahim Halil Aslan, Julie D. Pourtois, Veronica F. Frans, Meghan Forstchen, Maurice C. Goodman, Andrew J. Chamberlin, Kaitlyn R. Mitchell, Lorenzo Mari, Nathan C. Lo, Kamazima M. Lwiza, Nana R. Diakite, Mamadou Ouattara, Eliezer K. N’Goran, Chelsea L. Wood, Roseli Tuan, Fiona Allan, Roberta L. Caldeira, Antônio M.V. Monteiro, Jason Rohr, Erin A. Mordecai, Giulio A. De Leo

**Affiliations:** 1 Department of Biology, Stanford University, Stanford, CA, USA; 2 Department of Biology, University of Maryland, College Park, MD, USA; 3 Hopkins Marine Station, Stanford University, Pacific Grove, CA, USA; 4 Department of Biology, University of Notre Dame, Notre Dame, IN, USA; 5 School of Aquatic and Fishery Sciences, University of Washington, Seattle, WA, USA; 6 Department of Electronics, Information and Bioengineering, Politecnico di Milano, Milano, Italy; 7 Division of Infectious Diseases and Geographic Medicine, Department of Medicine, Stanford University, Stanford, CA, USA; 8 School of Marine and Atmospheric Sciences. Stony Brook University, New York, NY, USA; 9 Université Félix Houphouët-Boigny, 22 BP 770, Abidjan 22, Côte d’Ivoire; 10 Pasteur Institute, São Paulo Health Public Office, São Paulo, SP, Brazil; 11 Department of Life Sciences, Natural History Museum, London, UK; 12 René Rachou Institute, Fiocruz Minas - Belo Horizonte, MG, Brazil; 13 National Institute for Space Research, São José dos Campos, São Paulo, SP, Brazil; 14 Woods Institute for the Environment, Stanford University, Stanford, CA, USA

**Keywords:** Schistosomiasis, Infectious Diseases, Ecology, Seasonal temperature fluctuation, Climate change, Parasitic infection, Ectotherms

## Abstract

The complex relationship between temperature and schistosomiasis, an environmentally mediated neglected tropical disease affecting 250 million people globally, with hyperendemicity mostly in Africa, is poorly characterized. Here, we explored how seasonal temperature fluctuation affects the persistence, dynamics, and geographic distribution of schistosomiasis in Africa. We used a temperature-sensitive, mechanistic model of schistosomiasis dynamics that accounts for the adaptive behaviors of intermediate snail hosts and derived the disease’s thermal response curve for different patterns of seasonal temperature fluctuations. Changing the amplitude of seasonal temperature fluctuations can influence both the thermal optimum and critical thermal thresholds which imply accurately drawing the thermal response curves requires accounting for seasonality in addition to mean annual temperature. Moreover, our simulations can reproduce the documented persistence of schistosomiasis at locations with strong seasonal temperature fluctuations and mean annual temperatures near or above the critical thermal maxima for snail hosts only when snail adaptive behavior (e.g., aestivation, movement into cooler depths or shade) is included in the model. These results suggest that future climate change impacting the amplitude and timing of these fluctuations will likely alter the future geographic distribution of schistosomiasis in African regions. Our work demonstrates that a comprehensive understanding of schistosomiasis and, potentially, other environmentally mediated diseases in Africa, necessitates the inclusion of seasonal temperature fluctuations and host behavioral adaptations in process-based mechanistic models.

## Introduction

Many environmental factors that influence organismal performance, such as temperature and precipitation, exhibit cyclic and largely predictable seasonal fluctuations that exert strong pressures on the dynamics of environmentally mediated infectious diseases. These diseases are caused by pathogens or parasites with an important environmental component in their transmission cycle, such as vector- or waterborne diseases and soil-transmitted helminthiases. As ectotherms, the free-living stages of pathogens, parasites, and most vectors are unable to physiologically control their temperature. Consequently, ambient temperatures and their fluctuations^1–3^ can affect the transmission of environmentally-mediated diseases across multiple spatial and temporal scales^4–6^. Many diseases of conservation, veterinary, or public health importance are known for their strong seasonal dynamics^7^. A vast body of literature has investigated the effect of seasonal fluctuations on human diseases, such as influenza^8^ and measles^9^, or wildlife diseases, such as rabies^10^. Most of these studies^11^ have mostly modeled seasonal pathogen transmission rates or seasonal abundance of non-human reservoirs by using time-dependent functions rather than directly incorporating seasonal temperature. A study by^12^ developed a temperature-dependent model to explore the effects of seasonal temperature variation on mosquito-borne diseases.

Recent applications of thermally sensitive mechanistic modeling approaches assume that thermal performance curves (TPCs), derived at constant temperatures can be used to understand performance at variable temperatures such as through nonlinear averaging^13^. However, laboratory studies and theoretical analyses have shown that organisms’ thermal performance–and, specifically, their thermal optima, critical thermal maximum/minimum thresholds, and thermal breadth/safety margins–are strongly affected by daily, seasonal, and interannual temperature variations^6,14–19^. In some cases, piecewise functions for monthly temperatures, for example, may be appropriate for modeling diseases with short generation times (i.e., the average time from infection in a primary case to infection in secondary cases). However, for pathogens, parasites, and invertebrate hosts requiring longer timespans (e.g., >1 month) to complete their life cycles, this approach would underestimate the compound effects of long developmental time lags (e.g., vector or vertebrate reservoir age at reproductive maturity), especially in highly non-linear disease models.

Schistosomiasis, for example, is an environmentally mediated parasitic disease that has a complex life cycle, lasting over two months. It affects more than 250 million people worldwide, with the vast majority in Africa^20^. It is caused by blood flukes of the *Schistosoma* genus. Temperature variation affects *Schistosoma* eggs, their subsequent free-living stages (miracidia and cercariae), and their obligate intermediate snail hosts^21,22^. For instance, *Biomphalaria* snails are unable to lay eggs at temperatures below 15°C and above 30°C, while *Bulinus* snails cannot do so below 12.5°C and above 35°C^23–25^. In addition, higher temperatures result in a rapid rise in mortality for both *Biomphalaria* and *Bulinus* snails. Also, empirical studies indicate that the mortality rates of miracidia and cercariae increase rapidly when temperatures exceed 25°C^26–29^. However, miracidia infectivity also increases with temperature, until it reaches an inflection point and starts to sharply decay at temperatures above 30°C^30–32^. Therefore, extreme temperatures not only inhibit snail development, but also *Schistosoma* transmission.

Despite poor snail development at high temperatures, field studies show high prevalence of schistosomiasis in some regions, like Misungwi district, with temperatures above 30°C during some periods of the year. Higher temperatures might increase evaporation rate, leading to a faster depletion in freshwater like streams, ponds, and wetlands that completely dry up in the dry season.^33–41^. In such cases, some snail species can go into adaptive behavior stages like aestivation (or dormancy), where they burrow deep into the mud of desiccated ephemeral ponds, or moving to deeper water that remains cooler^42–45^. While a few studies of schistosomiasis account for seasonal variations in snail abundance^11,46^, most models omit two critical factors: the impacts of seasonal temperature fluctuations and snail adaptive behaviors. Thus, the effects of temperature fluctuations on *Schistosoma* transmission and the adaptive survival strategies of snails under extreme conditions for highly endemic schistosomiasis regions remain poorly studied.

Here, we built upon a thermally sensitive mechanistic model^47^ to simulate disease dynamics for increasing strengths of seasonal temperature fluctuations (i.e., differences between mean temperatures of warmest and coldest quarters; hereafter referred to as “seasonality”). We carefully calibrated the model for *Biomphalaria-S.mansoni* and *Bulinus-S.haematobium*—two major snail genera and parasite species pairs, respectively—to simulate and infer the seasonal oscillation of schistosome infections. Although the hydrological dynamics that shapes water habitats—critical for the survival and reproduction of snails—are inherently complex, temperature alone may provide a sufficient framework for understanding the complex ecological interactions like snails’ adaptive behaviors. Thus, we extended the model to account for snail adaptive behaviors as a function of temperature to better capture the historical spatial distribution of schistosomiasis in regions of Africa where MAT is close to or above schistosomiasis’ upper thermal limit. We then simulated schistosomiasis prevalence across Africa using a constant MAT derived from location-specific bioclimatic variables, alongside a 33-year historical daily temperature time series for each location to compare simulation outcomes under constant versus varying temperature conditions. Our findings demonstrate that incorporating seasonality and snail adaptive behaviors markedly influences the critical thermal limits, permitting persistence under variable temperature regimes that would be prohibitive under constant temperatures, while also improving the overall fit of the model to real-world data.

## Results

We simulated schistosomiasis dynamics with and without snail adaptive behaviors and for three levels of seasonality (ε = 0; 0.1; 0.25, which describe increasingly larger differences between the means of the warmest and coldest quarter of year), spanning the observed range of no, low, or high seasonality across a range of MAT in Africa. We compared model simulations against Global Neglected Tropical Diseases (GNTD) field data for runs using constant temperature, seasonality, and seasonality combined with snail adaptive behaviors. We found that both seasonality and snail adaptive behaviors altered the thermal response curve for schistosome prevalence. Accounting for seasonality shifted the TPC to the left towards lower temperatures, while including adaptive behaviors in combination with seasonality extended its thermal breadth and shifted the optimal temperature for schistosome transmission. In addition, simulations using both seasonality and snail adaptive behaviors gave the results most consistent with the GNTD data.

### Impacts of seasonality

**Figure 1:**
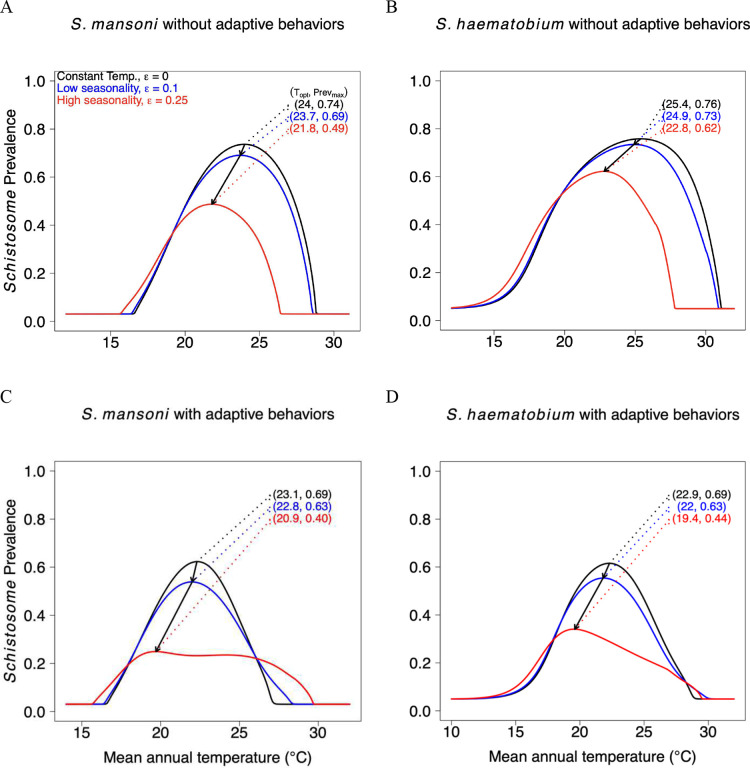
Seasonality and adaptive behaviors have distinct effects on the thermal response curve of schistosome prevalence. Simulations of schistosome prevalence for *S. mansoni* (A, C) and *S. haematobium* (B, D) across mean annual temperatures (MAT) for three seasonality values. Panels A and B show schistosome prevalence without snail adaptive behavior, while Panels C and D show schistosome prevalence with adaptive behaviors.

Simulations without seasonality (i.e., constant temperatures) generally resulted in higher schistosome prevalence, optimal, and critical temperatures for both *S. mansoni* and *S. haematobium* (Figure 1). Simulations with seasonality and without snail adaptive behaviors (Figure 1 A–B) showed a reduction in schistosome prevalence near and above the thermal optimum *T*_*opt*_ (temperature at peak prevalence), shifting the TPC – and, specifically, *T*_*opt*_, the critical thermal maximum *CT*_*max*_, and the critical thermal minimum *CT*_*min*_ – towards lower temperatures, and also reduced their thermal breadths (the difference between *CT*_*max*_ and *CT*_*min*_, Table 1). This occurs because the TPC is concave-down and left-skewed, so that temperature fluctuations near the optimum and above it tends to reduce performance compared to constant temperatures, whereas fluctuations near the thermal limit (where the TPC is locally concave-up) increase performance—an example of Jensen’s inequality.

### Impact of adaptive behaviors

When simulations included both seasonality and snail adaptive behaviors where we assume snails reduce their physiological activity and stop shedding the cercaria, there was a strong reduction in schistosome prevalence near and above the thermal optimum *T*_*opt*_, but the thermal response curve broadened and increased near the critical thermal limits (Figure 1 C–D). When we varied seasonality *ε* from 0 to 0.25, without adaptive behaviors *T*_*opt*_ had ranges from 21.8 °C to 24 °C for *S. mansoni* and from 22.8 °C to 25 °C for *S. haematobium*. Including adaptive behaviors changed these ranges to [23.1, 20.9] and [22.9, 19.4]. In addition, the critical thermal minimum *CT*_*min*_ shifted towards lower temperatures, while *CT*_*max*_ shifted towards higher temperatures, leading to an extension of the thermal breadth (Table 1).

### Model comparison

**Figure 2:**
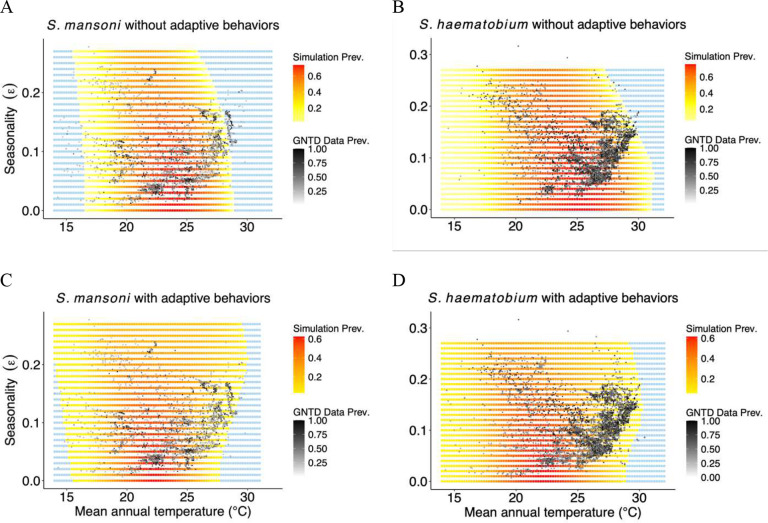
Models with seasonality and adaptive behaviors improve the fit to GNTD data. Comparison of simulated and observed schistosome prevalence for *S. mansoni* (A and C) and *S. haematobium* (B and D). Simulated values in panels A and B exclude snail adaptive behaviors while those in panels C and D include snail adaptive behaviors. Black circles represent observed prevalence from GNTD data, red and yellow squares indicate simulated prevalence. If the values of mean parasite burden below 0.75 for *S. mansoni*, and 0.3 for *S. haematobium*, it is assumed to be zero and shown in light blue.

We compared schistosome prevalence predicted by the models (constant temperature and seasonality, with and without snail adaptive behaviors) with field observations from 7,800 unique geographic locations where Schistosoma transmission has been historically reported. We found that including snail adaptive behaviors as a function of temperature was more consistent with field observations compared to other models (with the set of parameters for the adaptive behavior function (23, 0.085, 0.9) for *S. mansoni* and (23, 0.065, 0.7) for *S. haematobium)*. We calculated the Akaike Information Criteria (AIC) value for all three models (see Supplementary Material (SM)). For the *S. haematobium*, the model with adaptive behavior resulted the AIC score of 212,050.7, whereas the model with seasonal temperature but without adaptive behavior had a substantially higher AIC score of 673,675.8, and finally the model with constant temperature exhibited the highest AIC value of 1,502,795. Similarly, for *S. mansoni*, the AIC values were 218,688.6 for the model with adaptive behavior, 920,650.5 for the model with seasonal temperature but without adaptive behavior, and 876,934.2 for the model with constant temperature. These results indicate that incorporating adaptive behavior significantly improves model performance compared to models that rely solely on seasonal or constant temperature assumptions. Also, the reduction of *CT*_*max*_ and thermal breadth predicted by the model with only seasonality was at odds with the patterns of observed schistosome prevalence in many of the locations in the GNTD dataset (Figure 2 A–B). These data show a positive correlation between the degree of seasonality and disease persistence at increasing MATs (black dots in Figure 2), indicating that *CT*_*max*_ may increase with seasonality. This causes an increase in false negative rate for models with seasonality but without adaptive behavior, which decreases when snail adaptive behaviors are included. We found the false negative rates drop dawn from 0.335, to 0.25 for *S. mansoni* and from 0.57 to 0 for *S. haematobium* when we included the adaptive behaviors. And also, the model including snail adaptive behaviors was able to reproduce the observed pattern in disease prevalence, i.e., an increase in schistosome persistence at high MATs for increasing levels of seasonality (Figure 2 C–D, Table 1).

### Projections of prevalence differ among model assumptions

**Figure 3:**
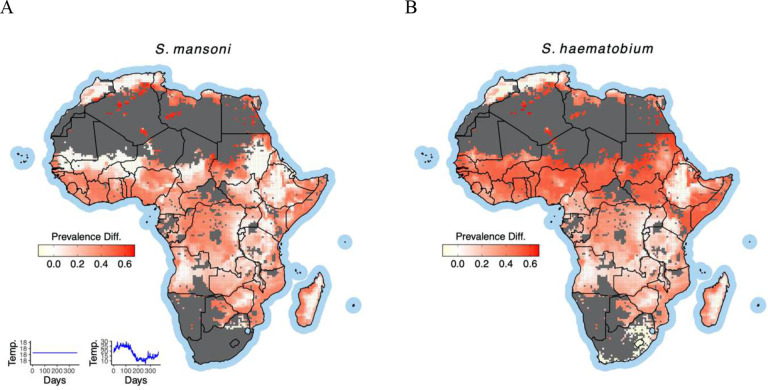
Model projections of difference in schistosome prevalence under constant and seasonality temperatures (shown in the subplot left corner) with adaptive behaviors differ across African regions for S. mansoni (A) and S. haematobium (B). The dark gray regions are masked out because they are not suitable for schistosomiasis for reasons other than temperature (distance from waterbody above 25 km, human population density below 2 individuals km^−2^).

Geographic projections of schistosome prevalence based on thermal suitability and persistence exhibit notable differences (Figure 3) when comparing simulations of the model using constant temperature to others incorporating seasonality with adaptive behaviors. These differences also vary between *S. mansoni* and *S. haematobium*, highlighting the role of species-specific ecological dynamics. Our simulations reveal that models assuming constant temperature tend to overestimate schistosome prevalence across nearly two-thirds of Africa. This overestimation is particularly pronounced in regions such as Liberia, Côte d’Ivoire, and Ghana for both *S. mansoni* and *S. haematobium*, suggesting that seasonality plays a significant role in shaping disease dynamics in these areas. In contrast, projections for countries like Tanzania, Kenya, and Ethiopia show minimal impact from incorporating seasonality. Interestingly, for some parts of the Sahel region, including Mali and eastern Gambia, the influence of seasonality on projections of *S. mansoni* prevalence is negligible compared to *S. haematobium*. While rare, there are also instances where constant-temperature models underestimate schistosomiasis prevalence, most notably in parts of South Africa (Figure 3).

## Discussion

We investigated the impact of temperature seasonality on schistosome transmission by using a thermally sensitive, mechanistic model that included adaptive behaviors of snails for extreme temperatures. Compared to constant temperatures, simulations with seasonality and without snail adaptive behaviors reduced peak schistosome prevalence, increased schistosome prevalence in the lower range of suitable temperatures and shifted the thermal optimum and the critical thermal limits for transmission to lower temperatures. Simulations with seasonality that also included the adaptive behavior of snails further flattened the thermal response curve and extended the critical thermal limits. Our results are compatible with observed data from 7,800 unique locations where schistosomiasis has been reported, showing that schistosomiasis may persist in regions with MAT well above 26–27°C (the predicted thermal limit under constant temperatures) as long as seasonality is high enough. These results also imply that using constant temperatures may overestimate schistosome prevalence for some regions whose MATs are near the optimal temperature for schistosomiasis. In contrast, simulations using constant temperature underestimate schistosome prevalence for other regions whose MATs are either very low or very high. Our modeling work provides support for the hypothesis that both seasonality and snail adaptive behaviors affect the temperature suitability of schistosome transmission across geographic regions, and that this effect can differ depending on the regions’ MAT. For instance, schistosomiasis may persist in regions with high seasonality and high MAT, which would not be predicted under models using constant temperatures.

Discrepancies between models with and without seasonality and seasonality with adaptive behavior can be traced back to Jensen’s inequality for concave/convex functions^14,48^ and the ecological and physiological responses of snails. The average of a nonlinear function’s value at two points will always be lower/higher than the function of the average of these two points. In the context of TPCs, this means that simulation of models based on average temperatures will not produce the same outcome as the simulation of a model including seasonally varying temperatures^14,48,49^. In addition, the adaptive behavior of snails to extreme temperatures directly influences their survival, reproduction, and parasite-host interactions^11,33–37,40,50,51^ all of which are critical components of schistosome transmission, changing the outcome of the models. Without accounting for these two details, models risk underestimating or overestimating the transmission potential^52^ and fail to capture the GNTD data trend, particularly in regions experiencing significant temperature variability or extreme climatic conditions. For instance, under constant temperatures above 31°C, several empirical studies reported extremely high snail mortality rates^24,25^, yet field observations found significant densities of snails and cercariae in ponds in Misungwi, district in Tanzania, where monthly mean temperatures reached as high as 35°C^35^. Our work shows that MAT, seasonality, and snail adaptive behaviors together can accurately capture the complexity of schistosome transmission.

Accurately defining the thermal envelope for schistosomiasis transmission is essential, as even small temperature variations can significantly influence transmission dynamics and human infection risk, as demonstrated in our simulations. With climate change driving global shifts in temperature patterns, precise knowledge of these thermal thresholds is crucial for predicting changes in endemic regions and implementing targeted control strategies. For instance, molluscicide application to reduce snail populations is most effective when timed to coincide with peak snail abundance^11^, which is largely temperature-driven^53^. Similarly, optimizing the timing of mass praziquantel (PZQ) administration based on regional temperature variability could enhance treatment efficacy and reduce disease burden. Reliable projections of schistosomiasis distribution, as well as assessments of its global burden under future climate scenarios, also require robust thermal modeling that incorporates seasonality. A comprehensive understanding of the thermal envelope governing schistosome transmission is thus fundamental for projecting the disease burden and designing region-specific interventions that effectively control and mitigate disease spread.

While our analysis offers improvements for predicting schistosome transmission, it nevertheless has some limitations. Our first limitation relates to the use of temperature seasonality as a proxy for snail adaptive behavior. Detailed empirical studies on snail adaptive behaviors are still relatively scarce and there is limited information available to parameterize these processes in disease models^43,54–56^. We assumed that snails transitioning into seasonal adaptive behaviors in response to higher temperatures. Such adaptive behaviors could include complex behaviors like aestivation, but it could also include simple behaviors such as movement within the water column or using microhabitats to behaviorally thermoregulate. Field observations suggest that it is the drying of water bodies (typically temporary ponds) during the drought season that primarily induces this adaptive behavior, and not temperature seasonality^57^. However, our correlation analysis between air temperature and precipitation, derived from a 30-year time series of ERA5 monthly data from 14,596 locations across Africa, shows that the correlation is negative in more than two-thirds of the locations (SM). Such findings support our use of air temperature as a proxy of environmental stress like water scarcity (i.e., drought) and resource limitation on snail vectors.

Our second limitation concerns model assumptions. We used a broader term for adaptive behaviors, where we assumed that schistosomiasis transmission would stop. It remains uncertain whether snails that migrate to deeper water layers (3–5 meters or more) and cercaria fully stop contributing to transmission, given that cercariae exhibit negative buoyancy^58,59^. However, assuming that strong thermal and density stratifications in lakes and rivers create significant physical barriers, it is highly challenging for cercariae to ascend from deeper, cooler waters to the surface. Therefore, it is reasonable to assume that while this behavioral response may enhance snail survival, it temporarily disrupts transmission dynamics.

Our third model limitation concerns the scale of our study. We were able to simulate disease transmission using solely temperature seasonality as a variable because our model was at the continental scale. At smaller scales, however, other factors such as soil type, size, depth and pond morphology, humidity, cloudiness, wind could impact model performance, and thus may need to be included in future, smaller-scaled studies. Simulating these processes requires detailed complex hydrological-climatic models, fine-tuned over specific water bodies in each region and the result of such analysis would be either computationally costly or location specific.

Like many other infectious diseases, schistosomiasis transmission is likely influenced by seasonal temperature variation, and the intermediate host’s behavioral response to it. Using mechanistic models, we found that seasonal temperature variation reduces the predicted optimal temperature and upper thermal limits, while adaptive behaviors of snails can extend the thermal range for transmission. These findings emphasize the necessity of considering seasonality and snail behavioral adaptations to better understand schistosomiasis transmission, as these processes significantly enhance the accuracy of geographical projections of disease prevalence and persistence.

## Methodology

### Model

**Figure 4:**
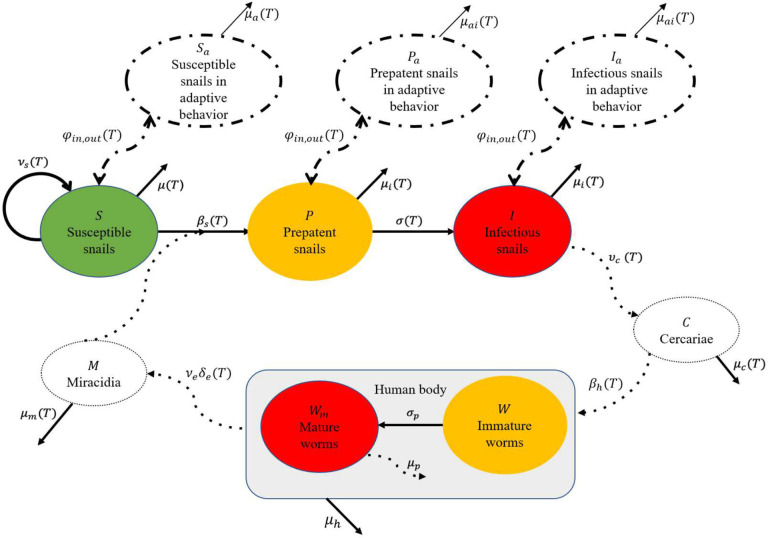
Compartmental diagram. Susceptible snails reproduce and die at rates *v*_*s*_(*T*), *μ*(*T*), respectively or transition to prepatent snails *β*_*s*_(*T*) factor with density of miracidia. Prepatent snails either die or become infectious at rates *μ*_*i*_(*T*) and *σ*(*T*). Infectious snails die at rate *μ*_*i*_(*T*) and shed cercariae at rate *v*_*c*_(*T*). Susceptible, prepatent, and infectious snails transition to adaptive behaviors stages and revert from them at rate *φ*_*in*,*out*_ (*T*). During adaptive behaviors, susceptible snails die at rate *μ*_*a*_(*T*), while prepatent and infectious snails die at rate *μ*_*ai*_(*T*). Cercariae either die at rate *μ*_*c*_(*T*) or penetrate human skin at rate *β*_*h*_(*T*). Immature Schistosoma parasites become mature at rate *σ*_*p*_. Mature parasites die at rate *μ*_*p*_ or die due to human death with rate *μ*_*h*_ and produce number of eggs *v*_*e*_. These eggs hatch with probability of success *δ*_*e*_(*T*), becoming miracidia, which either die at rate *μ*_*m*_(*T*) or invade susceptible snail tissue, thus closing the transmission cycle.

We built upon a temperature-sensitive mechanistic model of *Schistosoma* spp. infection^47^ to simulate the dynamics of schistosomiasis with seasonally varying temperatures. The model tracks the abundance of susceptible (*S*), prepatent (infected but not shedding yet, *P*), and infectious (shedding, *I*) snails, as well as the mean parasite burden for immature (*W*) and mature (sexually reproductive, *W*_*M*_) parasites in the human population (Figure 4).

The model incorporates key thermal-sensitive demographic and epidemiological parameters, including the snails’ per capita fecundity, mortality, and prepatent rates; the miracidial hatching success and mortality rates, the per-capita daily cercarial shedding and mortality rates; and the probabilities of infection, all as functions of temperature. Additionally, the model includes temperature-independent parameters, such as the maturation rate of parasites, the egg production rate of worms, and the mortality rates of humans and parasites^60^. For a detailed description of the parameters see^47^ (see SM).

### Inclusion of snail’s adaptive behavior

We assumed that snails migrate to temperature favorable environments to optimize their survival and thus enhanced our model by introducing snail adaptive behaviors. As temperatures rise, snails may either retreat to deeper water or enter a state of aestivation depending on the water bodies they are in. These migrations limit snails’ access to resources, leading to a reduction in key life processes, such as reproduction. We further assumed that during these periods of retreat or aestivation, snails are less likely to shed cercariae, and any cercariae released may struggle to reach the water surface. This adaptive behavior essentially slows down disease transmission, so we assumed that snails in adaptive behavioral stages do not reproduce or shed cercariae, and that their mortality rates are 20x lower than for susceptible (*S*) and 2x lower than for infected (*P*, *I*) snails, respectively, according to previous studies^43,54–56^.

Most studies on snail adaptive behaviors such as aestivation consider precipitation as the main driver of their adaptive behavior. However, here, we used temperature as a proxy of environmental stress instead of precipitation because we found some correlation between temperature and precipitation. We examine the broader patterns of correlation between temperature and precipitation across African regions We retrieved a 30-year time series of ERA5 mean monthly temperature and precipitation data for the entire African continent. To account for spatial autocorrelation, we averaged the data over 10 × 10 km (100 km2) quadrats, systematically distributed 50 km apart across Africa. Finally, we computed the correlation between temperature and precipitation for the 360 monthly observations at each of the 14,596 unique locations (see SM). We used an S-shaped (sigmoid) function of temperature *T* to describe the transition both to and from across three adaptive behavior compartments of snails, (dash-dotted in): susceptible (*S*_*a*_), prepatent (*P*_*a*_), and infectious (*I*_*a*_), as follows:

$$\varphi_{\text{in}}\left(T\right)=\frac{e_{o}}{1+e^{-\alpha \left(T-T_{o}\right)}},\;\varphi_{\text{out}}\left(T\right)=\frac{e_{o}}{1+e^{\alpha \left(T-T_{o}\right)}}$$

Here, the parameter *T*_*o*_ is the temperature at which *φ*_*in*_(*T*) and *φ*_*out*_(*T*) reach half of their maximum saturation value *e*_*o*_ and *α* is inversely related to the steepness of the transitioning function of temperature. We used the maximum likelihood estimator with the GNTD dataset for the best set of parameters (*e*_0_, *T*_0_, *α*) (see SM).

### Simulations

To project thermal suitable map of schistosomiasis (*S. mansoni* and *S. haematobium*) prevalence for Africa, we used 50×50 km grid, chosen to balance resolution and computational efficiency, then run the models with daily mean temperatures of each grid from 1990 to 2023. We took the mean of MPB (Mean Parasite Burden) from the final five years of the mature parasite burden compartment and converted this value to the prevalence of schistosomiasis in the population by using the probability of having one or more worms, following^61^ and^62^ (see SM). Regions unsuitable for schistosomiasis—those located more than 25 km from freshwater or with population densities below 2 people per square kilometer—were masked, following criteria established in^47^.

To derive thermal response curve across three different seasons, we used a smooth sinusoidal function of time:

$$T\left(t\right)=T_{\text{med}}\left(1+\varepsilon \;\text{sin}\left(\frac{2\pi t}{365}\right)\right)$$

to produced temperature time series. Here  *T*_*med*_ represents the mean annual temperature (MAT) and *ε*  is the magnitude of seasonality, reflecting the scale of temperature variation over a year. Notably, this function produces the same temperature for a given day of the year, regardless of the year, with minimal day-to-day variation, resulting in a smooth and predictable temperature trajectory for each month. We ran the model with this sinusoidal time dependent temperature function for 40 years to reach an endemic condition and then like above we converted the mean of MPB obtained from the final five years to the prevalence of schistosomiasis. We used three seasonality values *ε* (constant, low, and high) which are chosen to cover the range of the seasonality values across Africa to compare the impact of seasonal intensity on the thermal response curve.

To demonstrate the models’ outcomes with GNTD data, we ran the model with sinusoidal function for the *ε* ∈ [0.02, 0.26] with increasing step 0.01 and *T*_*med*_. ∈ [14, 31] with increasing step 0.2. We also extracted bioclimatic variables for each GNTD data locations from WorldClim: the mean temperature of the warmest quarter (BIO10), the mean temperature of the coldest quarter (BIO11), and MAT (BIO01), following^63^ and use the following formula to calculate seasonality value for each GNTD data (see SM):

$$\varepsilon =\frac{\left(\text{BIO}10-\text{BIO}11\right)\pi }{4\sqrt{2}\;\text{BIO}01}$$

and then plot *ε* versa *T*_*med*_. with the prevalence of models ‘outcome and GNTD data together.

The initial conditions for all our simulations: 46,000 susceptible snails; 1,200 prepatent snails; 2,300 infectious snails; 3 *S. mansoni* and 1 *S. haematobium* immature mean parasites burden; 130 *S. mansoni* and 45 *S. haematobium* mature mean parasites burden. The mean parasite burden is expressed per 1,000 people.

### Model comparison

We calculated AIC score for three models (the model with adaptive behavior, the model with seasonal temperature but without adaptive behavior and the model with constant temperature) by assuming the distribution of schistosomiasis in the population is binomial distributed. We compare the models’ performance based on these AIC scores (see SM).

## Figures and Tables

**Table 1:** Critical thermal maximum and minimum for the model with and without snail adaptive behaviors for different amplitudes of seasonal temperature fluctuations.

Species	Models	No seasonality (*ε* = 0) [*CT*_*min*_, *CT*_*max*_]	Low seasonality (*ε* = 0.1) [*CT*_*min*_, *CT*_*max*_]	High seasonality (*ε* = 0.25) [*CT*_*min*_, *CT*_*max*_]
***S. mansoni***	No adaptive behaviors	[16.5, 28.9] ° C	[16.3, 28.6] ° C	[15.5, 26.5] ° C
	Adaptive behaviors	[16.4, 26.7] ° C	[16.3, 28] ° C	[15.7, 29.7] ° C
***S. haematobium***	No adaptive behaviors	[13.9, 31.1] ° C	[13.7, 31] ° C	[13.1, 27.9] ° C
	Adaptive behaviors	[12.6, 29.2] ° C	[12.6, 30.5] ° C	[11.9, 29.4] ° C
